# Supplementary material for: Effect of Zwitterionic Additives on Solvation and Transport of Sodium and Potassium Cations in (Ethylene Oxide)10: A Molecular Dynamics Simulation Study
Source: Nanomaterials (Basel). 2024 Jan 19;14(2):219. doi: 10.3390/nano14020219 (PMC10818316; doi:10.3390/nano14020219)
Supplement: Supplementary file 1 [file nanomaterials-14-00219-s001.zip › nanomaterials-2742706-supplementary.pdf]

Supplementary materials

Effect of Zwitterionic Additives on Solvation and Transport of Sodium and Potassium Cations in (Ethylene Oxide)$_{10}$: a Molecular Dynamics Simulation Study

Manh Tien Nguyen[1,2*], Yuhua Duan[2], Qing Shao[1*]

[1] Chemical and Materials Engineering Department, University of Kentucky, Lexington, KY 40506, USA

[2] National Energy Technology laboratory, United States Department of Energy, Pittsburgh, PA 15236, USA

List of tables:

Table S1. Force field parameters. Labels as shown in Figure 1. 2

Table S2. Diffusion coefficient of Na^+/K^+ at 600K (nm^2/ns).. 6

Table S3. Diffusion coefficient of $[TFSI]^-$ at 600K (nm^2/ns)... 7

Table S4. Diffusion coefficients of ZW at 600K (nm^2/ns)... 7

Table S5. Coordination numbers of Na^+-O in systems at 353K 9

Table S6. Coordination numbers of K^+-O in systems at 353K.. 9

Table S7. Diffusion coefficient of Na^+/K^+ at 353K (10^{-4} nm^2/ns) 9

List of figures:

Figure S1. Molecular structures of (a) ChoPO4, (b) ImSO3, (c) ImCO2, (d) $[TFSI]^-$, and (e) EO_{10}. Every atom is labeled with a unique name. Atom color code: hydrogen (silver), carbon (cyan), nitrogen (blue), oxygen (red), sulfur (yellow). 4

Figure S2. Radial distribution functions of Na^+/ K^+-O(ZW) at 600K. Atom names are the same as in Figure 1. K^+ prefers to coordinate with two O atoms (O45) having double bonds with P atom on PO_4 group of ChoPO4.. 5

Figure S3. Snapshot of (a) 4 ChoPO4 coordinating with 4 Na^+, (b) 4 ImCO2 coordinating with 5 Na^+, and (c) 1 ImSO3 coordinating with 3 Na^+ in Na^+ system. Na^+ ions are represented using the VDW model with yellow colour; ZW molecules are displayed with the CPK model; $[TFSI]^-$ and EO_{10} molecules are displayed using the line model. Atom color code: hydrogen (silver), carbon (cyan), nitrogen (blue), oxygen (red), sulfur (yellow). 5

Figure S4. Snapshot of (a) 4 ChoPO4 coordinating with 4 K^+, (b) 4 ImCO2 coordinating with 5 K^+, and (c) 1 ImSO3 coordinating with 3 K^+ in K^+ system. K^+ ions are represented using the VDW model with ochre colour; ZW molecules are displayed with the CPK model; $[TFSI]^-$ and EO_{10} molecules are displayed using the line model. Atom color code: hydrogen (silver), carbon (cyan), nitrogen (blue), oxygen (red), sulfur (yellow). 5

Figure S5. Residence C(t) curves of (a) Na^+-O($[TFSI]^-$) in, (b) Na^+-O(EO_{10}) and (c) Na^+-O(ZW) in NaTFSI/EO_{10} systems at 600K. 6

Figure S6. Residence C(t) curves of (a) K^+-O($[TFSI]^-$) in, (b) K^+-O(EO_{10}) and (c) K^+-O(ZW) in NaTFSI/EO_{10} systems at 600K. 6

Figure S7. MSD curves of (a) Na^+, (b) $[TFSI]^-$ and (c) ZW in NaTFSI/EO_{10} systems at 600K. ... 6

Figure S8. MSD curves of (a) K^+, (b) $[TFSI]^-$ and (c) ZW in KTFSI/EO_{10} systems at 600K. 6

Figure S9. Distance travelled by Na^+ ions in a specific O coordination number over 2-ns for systems (a) with ChoPO4, (b) with ImCO2, and (c) with ImSO3. 7
Figure S10. Distance travelled by K^+ ions in a specific O coordination number over 2-ns for systems (a) with ChoPO4, (b) with ImCO2, and (c) with ImSO3. 7
Figure S11. Radial distribution functions of (a) Na^+/ K^+-O([TFSI]$^-$), (b) Na^+/ K^+-O(EO_{10}), (c) Na^+/ K^+-O(ZW) and (d) Na^+- Na^+ and K^+-K^+ in NaTFSI/EO_{10} and KTFSI/EO_{10}systems at 353K. 8
Figure S12. Radial distribution functions of cation-ZW in NaTFSI/EO_{10} and KTFSI/EO_{10} systems at 353K. 9
Figure S13. Residence C(t) curves of (a) Na^+-O([TFSI]$^-$) in, (b) Na^+-O(EO_{10}) and (c) Na^+-O(ZW) in c systems at 353K. Colors correspond to the presence of ZW molecules. 9
Figure S14. Residence C(t) curves of (a) K^+-O([TFSI]$^-$) in, (b) K^+-O(EO_{10}) and (c) K^+-O(ZW) in c systems at 353K. Colors correspond to the presence of ZW molecules. 9
Figure S15. Percentage of cations that are not coordinating with EO_{10} at 353K. 10
Figure S16. Rg distribution in (a) Li^+, (b) Na^+, (c) K^+ systems 10

Table S1. Force field parameters. Labels as shown in Figure S1.

Label	σ (nm)	ε (kJ/mol)	q (e)
ChoPO4			
C1	0.355	0.317984	-0.1723
C2	0.355	0.317984	-0.1605
C3	0.35	0.276144	-0.2016
C4	0.355	0.29288	0.4866
C5	0.35	0.276144	-0.0058
C6	0.35	0.276144	0.0849
C7	0.35	0.276144	-0.002
C8	0.35	0.276144	-0.0943
C9	0.35	0.276144	-0.1506
CX	0.35	0.276144	-0.1822
CY	0.35	0.276144	-0.1863
H11, and H12	0.25	0.12552	0.1428
H31, H32, and H33	0.25	0.12552	0.0983
H51, and H52	0.25	0.12552	0.1025
H61, and H62	0.25	0.12552	0.0971
H71, and H72	0.25	0.12552	0.0811
H81, and H82	0.25	0.12552	0.1306
H91, H92, and H93	0.25	0.12552	0.1608
HX1, HX2, and HX3	0.25	0.12552	0.1519
HY1, HY2, and HY3	0.25	0.12552	0.139
N	0.325	0.71128	-0.0123
O1	0.296	0.87864	-0.4613
O2	0.29	0.58576	-0.3565
O3	0.29	0.58576	-0.9069
O4, and O5	0.296	0.87864	-1.1686
O6	0.29	0.58576	-0.8125

P	0.374	0.8368	2.7126
ImCO2			
C	0.355	0.29288	0.486
C1	0.35	0.276144	-0.276
C2	0.35	0.276144	0.023
C3	0.355	0.29288	0.198
C4	0.355	0.29288	-0.074
C5	0.355	0.29288	-0.077
C6	0.35	0.276144	0.002
C7	0.35	0.276144	-0.171
C8	0.35	0.276144	-0.29
H11, H12, and H13	0.25	0.12552	0.119
H21, and H22	0.25	0.12552	0.135
H3	0.242	0.12552	0.347
H4	0.242	0.12552	0.234
H5	0.242	0.12552	0.242
H61, and H62	0.25	0.12552	0.145
H71, and H72	0.25	0.12552	0.107
H81, and H82	0.25	0.12552	0.088
N1	0.325	0.71128	-0.324
N2	0.325	0.71128	-0.213
O1, and O2	0.296	0.87864	-0.707
ImSO3			
C1	0.35	0.276144	-0.2595
C2	0.35	0.276144	-0.0352
C3	0.355	0.29288	0.0914
C4	0.355	0.29288	-0.1274
C5	0.355	0.29288	-0.08
C6	0.35	0.276144	-0.066
C7	0.35	0.276144	-0.133
C8	0.35	0.276144	-0.6807
H11, H12, and H13	0.25	0.12552	0.1196
H21, and H22	0.25	0.12552	0.1324
H3	0.242	0.12552	0.2719
H4	0.242	0.12552	0.25
H5	0.242	0.12552	0.2744
H61, and H62	0.25	0.12552	0.1318
H71, and H72	0.25	0.12552	0.1279
H81, and H82	0.25	0.12552	0.1329
N1	0.325	0.71128	-0.1837
N2	0.325	0.71128	-0.087
O1, O2, and O3	0.296	0.71128	-0.7122
S	0.355	1.046	1.4926
$[TFSI]^-$			
N	0.325	0.71128	-0.66

S1 and S2	0.355	1.046	1.02
O11, O12, O21 and O22	0.296	0.87864	-0.53
C1 and C2	0.35	0.27614	0.35
F11, F12 and F13	0.295	0.22175	-0.16
F21, F22 and F23	0.295	0.22175	-0.16
EO_{10}			
C1, and C20	0.35	0.276144	0.11
H1-3, and H40-42	0.25	0.12552	0.03
O1-10	0.29	0.58576	-0.4
C2-19	0.35	0.276144	0.14
H4-39	0.25	0.12552	0.03

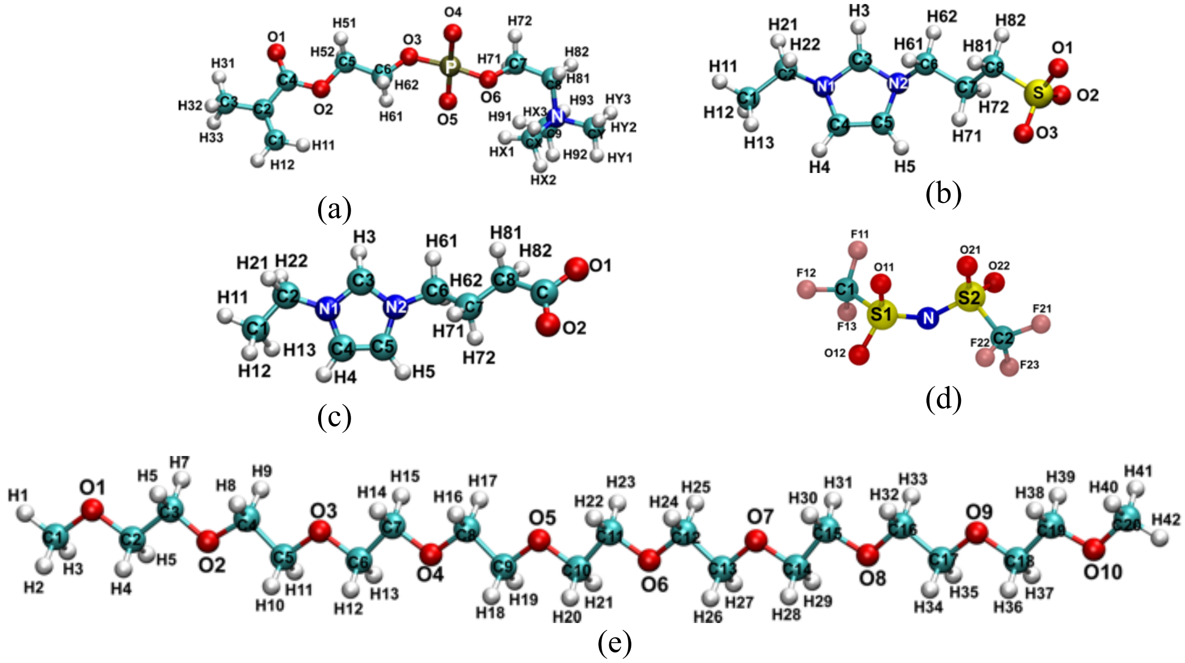

Figure S1. Molecular structures of (a) ChoPO4, (b) ImSO3, (c) ImCO2, (d) [TFSI]$^-$, and (e) EO_{10}. Every atom is labeled with a unique name. Atom color code: hydrogen (silver), carbon (cyan), nitrogen (blue), oxygen (red), sulfur (yellow).

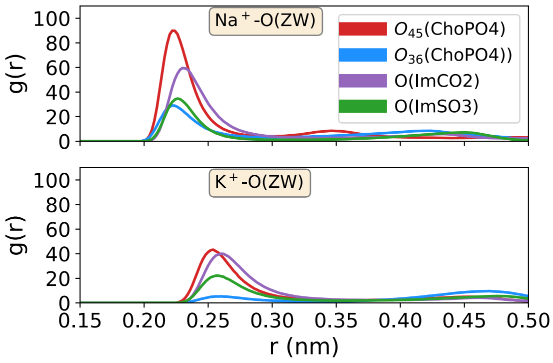

Figure S2. Radial distribution functions of Na^+/ K^+-O(ZW) at 600K. Atom names are the same as in Figure 1. K^+ prefers to coordinate with two O atoms (O45) having double bonds with P atom on PO_4 group of ChoPO4.

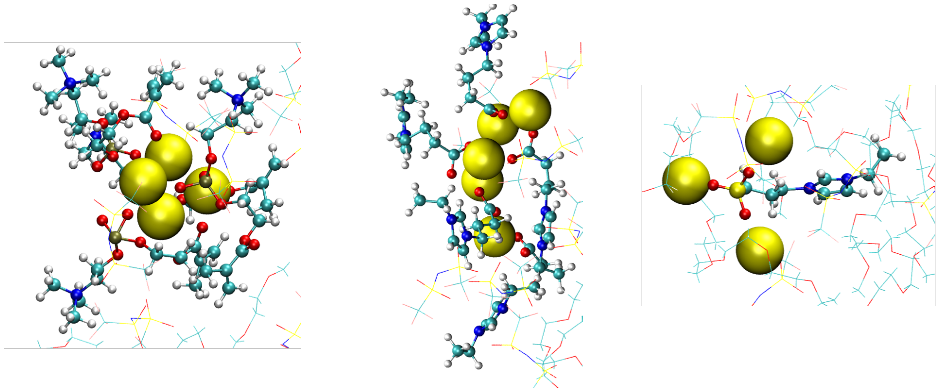

(a) (b) (c)

Figure S3. Snapshot of (a) 4 ChoPO4 coordinating with 4 Na^+, (b) 4 ImCO2 coordinating with 5 Na^+, and (c) 1 ImSO3 coordinating with 3 Na^+ in Na^+ system. Na^+ ions are represented using the VDW model with yellow colour; ZW molecules are displayed with the CPK model; $[TFSI]^-$ and EO_{10} molecules are displayed using the line model. Atom color code: hydrogen (silver), carbon (cyan), nitrogen (blue), oxygen (red), sulfur (yellow).

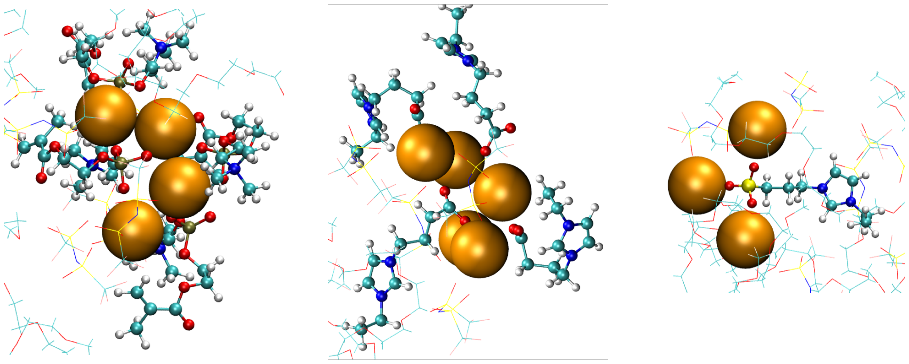

(a) (b) (c)

Figure S4. Snapshot of (a) 4 ChoPO4 coordinating with 4 K^+, (b) 4 ImCO2 coordinating with 5 K^+, and (c) 1 ImSO3 coordinating with 3 K^+ in K^+ system. K^+ ions are represented using the VDW model with ochre colour; ZW molecules are displayed with the CPK model; $[TFSI]^-$ and EO_{10} molecules are displayed using the line model. Atom color code: hydrogen (silver), carbon (cyan), nitrogen (blue), oxygen (red), sulfur (yellow).

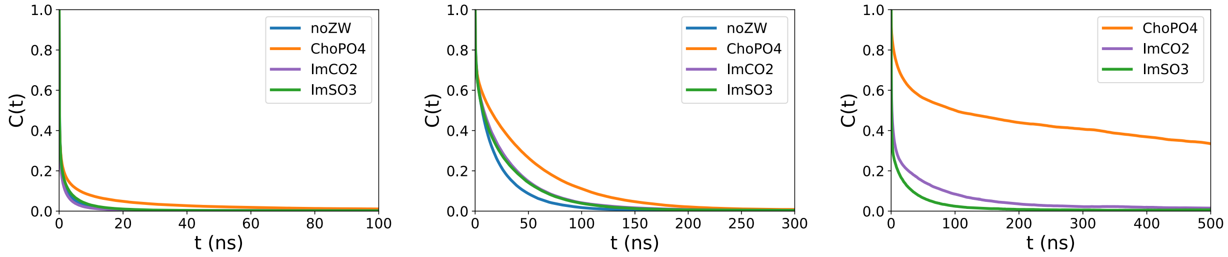

(a) (b) (c)

Figure S5. Residence C(t) curves of (a) Na^+-O($[TFSI]^-$) in, (b) Na^+-O(EO_{10}) and (c) Na^+-O(ZW) in $NaTFSI/EO_{10}$ systems at 600K.

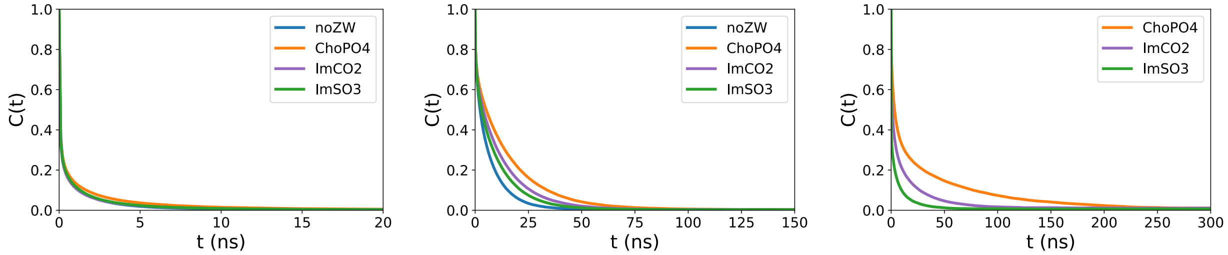

(a) (b) (c)

Figure S6. Residence C(t) curves of (a) K^+-O($[TFSI]^-$) in, (b) K^+-O(EO_{10}) and (c) K^+-O(ZW) in $NaTFSI/EO_{10}$ systems at 600K.

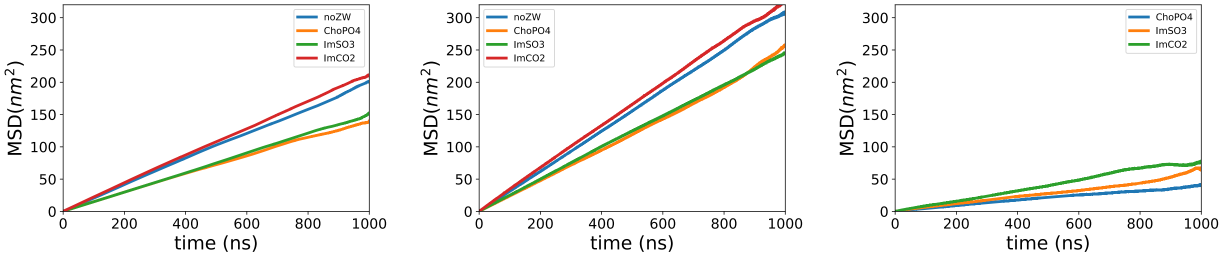

(a) (b) (c)

Figure S7. MSD curves of (a) Na^+, (b) $[TFSI]^-$ and (c) ZW in $NaTFSI/EO_{10}$ systems at 600K.

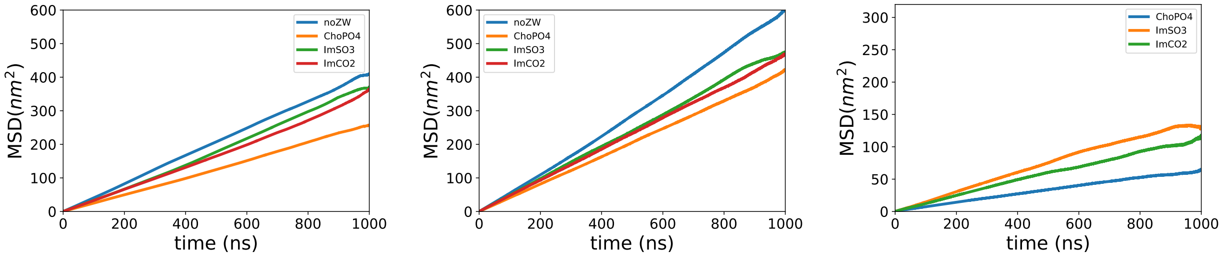

(a) (b) (c)

Figure S8. MSD curves of (a) K^+, (b) $[TFSI]^-$ and (c) ZW in $KTFSI/EO_{10}$ systems at 600K.

Table S2. Diffusion coefficient of Na^+/K^+ at 600K (nm^2/ns)

System	noZW	ChoPO4	ImCO2	ImSO3
Na^+	0.030± 0.003	0.028 ± 0.003	0.036 ± 0.001	0.027 ± 0.001
K^+	0.066 ± 0.008	0.041 ± 0.002	0.056 ± 0.002	0.056 ± 0.005

Table S3. Diffusion coefficient of $[TFSI]^-$ at 600K (nm^2/ns)

System	noZW	ChoPO4	ImCO2	ImSO3
Na^+	0.047 ± 0.004	0.041 ± 0.002	0.055 ± 0.006	0.042 ± 0.001
K^+	0.095 ± 0.004	0.067 ± 0.002	0.076 ± 0.001	0.077 ± 0.003

Table S4. Diffusion coefficients of ZW at 600K (nm^2/ns)

System	ChoPO4	ImCO2	ImSO3
Na^+	0.007 ± 0.001	0.012 ± 0.002	0.010 ± 0.002
K^+	0.010 ± 0.001	0.016 ± 0.004	0.025 ± 0.001

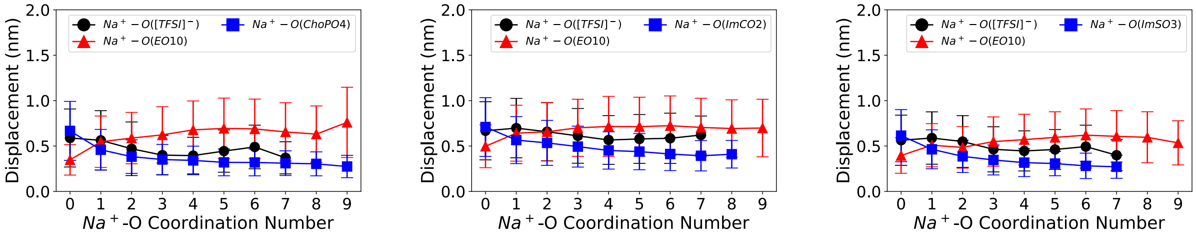

(a) (b) (c)

Figure S9. Distance travelled by Na^+ ions in a specific O coordination number over 2-ns for systems (a) with ChoPO4, (b) with ImCO2, and (c) with ImSO3.

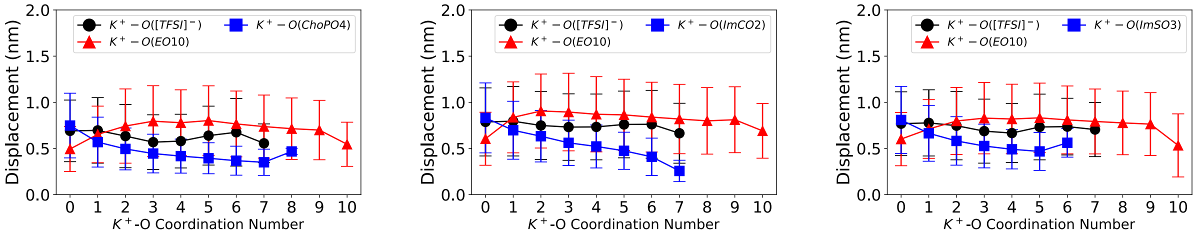

(a) (b) (c)

Figure S10. Distance travelled by K^+ ions in a specific O coordination number over 2-ns for systems (a) with ChoPO4, (b) with ImCO2, and (c) with ImSO3.

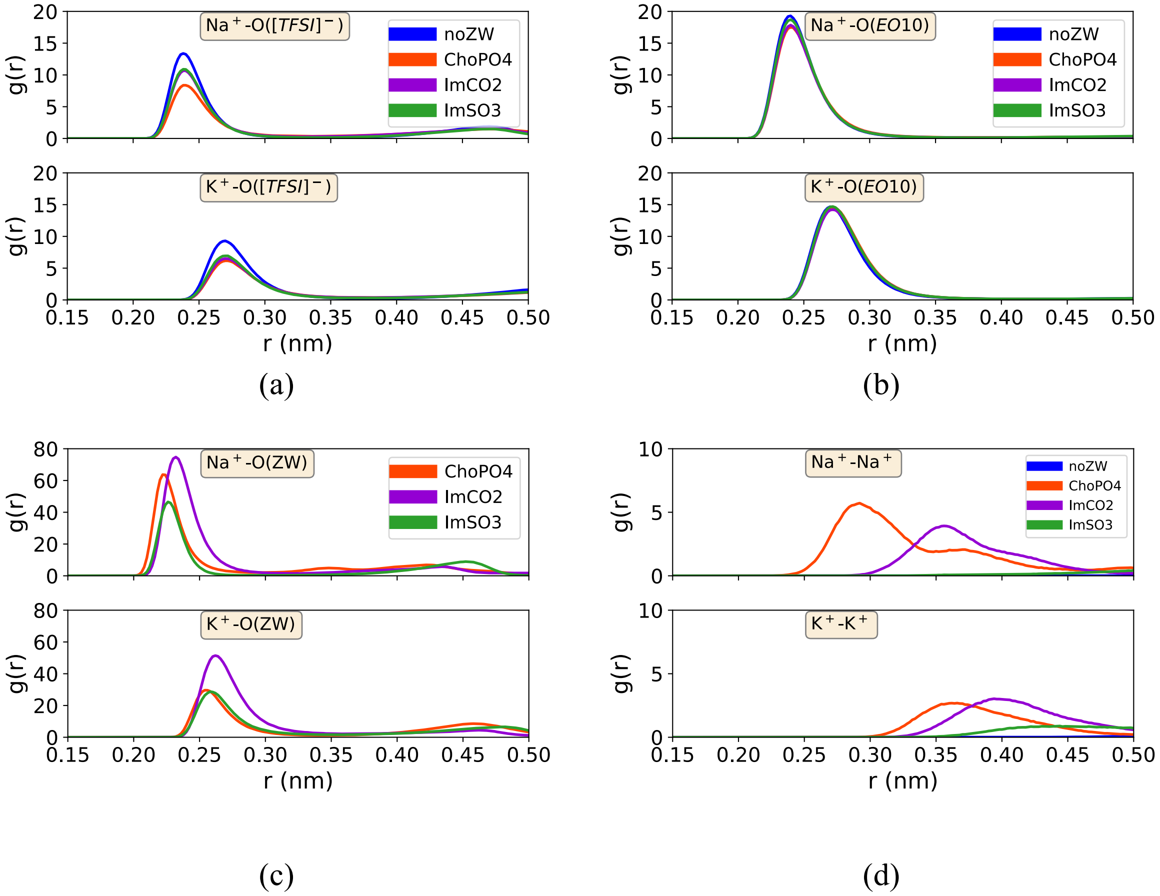

Figure S11. Radial distribution functions of (a) Na^+/ K^+-O($[TFSI]^-$), (b) Na^+/ K^+-O(EO_{10}), (c) Na^+/ K^+-O(ZW) and (d) Na^+- Na^+ and K^+-K^+ in NaTFSI/EO_{10} and KTFSI/EO_{10}systems at 353K.

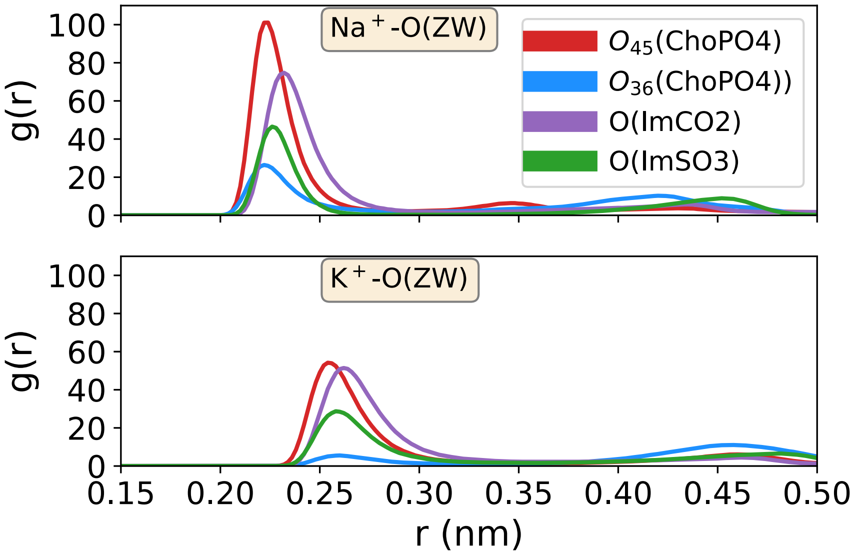

Figure S12. Radial distribution functions of cation-ZW in NaTFSI/EO_{10} and KTFSI/EO_{10} systems at 353K.

Table S5. Coordination numbers of Na^+-O in systems at 353K

	No ZW	ChoPO4	ImCO2	ImSO3
Total	7.18 ± 0.02	7.02 ± 0.01	7.10 ± 0.01	6.96 ± 0.01
Na^+-O(ZW)	N/A	1.20 ± 0.02	0.93 ± 0.03	0.62 ± 0.05
Na^+-O([TFSI]$^-$)	2.05 ± 0.03	1.43 ± 0.04	1.61 ± 0.03	1.62 ± 0.01
Na^+-O(EO_{10})	5.13 ± 0.05	4.39 ± 0.05	4.56 ± 0.02	4.72 ± 0.05

Table S6. Coordination numbers of K^+-O in systems at 353K

	No ZW	ChoPO4	ImCO2	ImSO3
Total	7.91 ± 0.01	7.83 ± 0.02	7.97 ± 0.01	7.88 ± 0.03
K^+-O(ZW)	N/A	1.05 ± 0.04	1.04 ± 0.02	0.86 ± 0.05
K^+-O([TFSI]$^-$)	2.24 ± 0.01	1.50 ± 0.04	1.62 ± 0.03	1.63 ± 0.01
K^+-O(EO_{10})	5.67 ± 0.01	5.28 ± 0.02	5.31 ± 0.01	5.39 ± 0.03

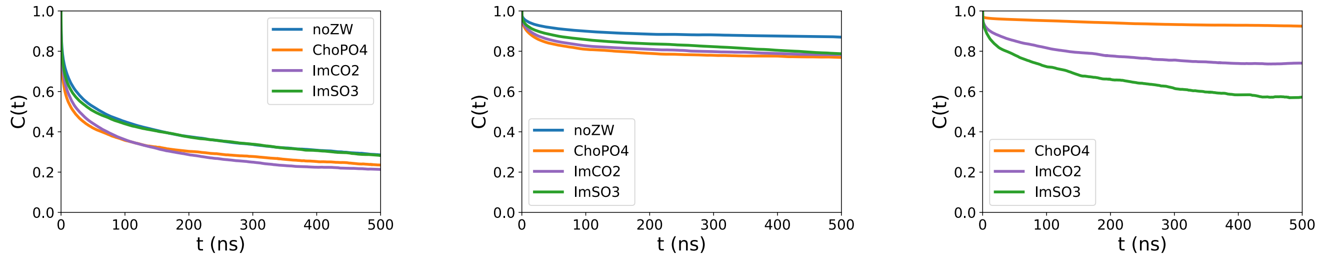

(a) (b) (c)

Figure S13. Residence C(t) curves of (a) Na^+-O([TFSI]$^-$) in, (b) Na^+-O(EO_{10}) and (c) Na^+-O(ZW) in c systems at 353K. Colors correspond to the presence of ZW molecules.

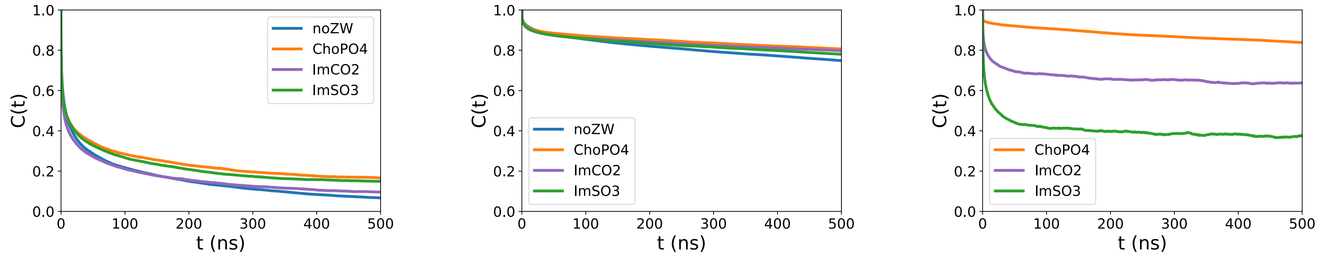

(a) (b) (c)

Figure S14. Residence C(t) curves of (a) K^+-O([TFSI]$^-$) in, (b) K^+-O(EO_{10}) and (c) K^+-O(ZW) in c systems at 353K. Colors correspond to the presence of ZW molecules.

Table S7. Diffusion coefficient of Na^+/K^+ at 353K (10^{-4} nm^2/ns)

System	noZW	ChoPO4	ImSO3	ImCO2
Na^+	2.1± 0.2	1.7 ± 0.01	2.4 ± 0.4	2.8 ± 0.4
K^+	0.63± 0.08	1.6 ± 0.09	2.4 ± 0.05	1.6 ± 0.3

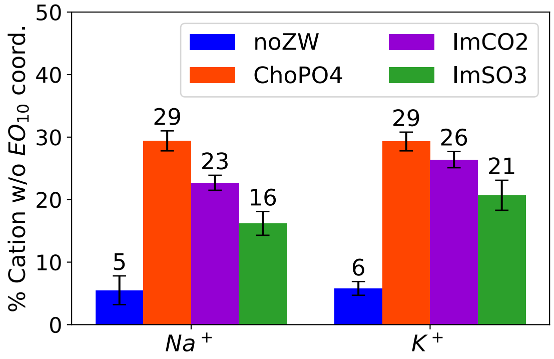

Figure S15. Percentage of cations that are not coordinating with EO_{10} at 353K.

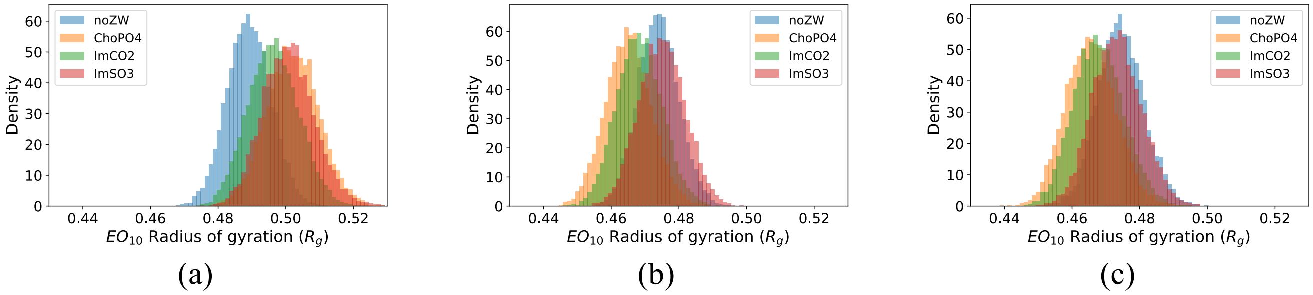

Figure S16. Rg distribution in (a) Li^+, (b) Na^+, (c) K^+ systems
